# The Rise of Independent Prescribing by Optometrists in Wales 2020–2024: Number of Practices, Drugs and Costs

**DOI:** 10.1007/s44402-026-00097-1

**Published:** 2026-05-04

**Authors:** David Grant Robinson, Hannah Thomas, Barbara Ryan

**Affiliations:** School of Optometry and Vision Sciences, Cardiff University, Cardiff, UK

**Keywords:** Independent prescribing, Optometrists, Optometry, Public health, Urgent eye care, Wales

## Abstract

**Purpose:**

To evaluate the growth and prescribing patterns of community-based independent prescribing (IP) optometrists managing acute eye conditions in Wales between 2020 and 2024, a period that saw the introduction and subsequent commissioning of National Health Service (NHS) funded acute eye care with prescribing in primary care optometry throughout Wales.

**Methods:**

Monthly prescribing data from NHS Wales Shared Services Partnership were analysed for all IP optometrists in Wales from 1st February 2020 to 31st January 2024. Data included drug name, British National Formulary classification, quantity, cost and health board location. Descriptive and correlational statistics were used to assess prescribing activity, regional distribution and cost trends.

**Results:**

The number of active IP optometry practices increased from eight in February 2020 to 68 in January 2024, with 20,980 prescriptions (49,162 items) issued at a total cost of £339,426. Corticosteroids, anti-infective agents and ocular lubricants were the most frequently prescribed drug classes. Ocular lubricants accounted for 34.0% of the total spend. Regional variation in prescribing activity was observed, with positive correlations between the number of active practices and both prescription volume and cost. Generic prescribing accounted for 47.0% of prescriptions, lower than national averages.

**Conclusion:**

NHS commissioning of IP services in Wales has significantly expanded the role of optometrists in managing acute eye conditions in primary care. The findings highlight the potential of IP optometry to reduce pressure on general medical practice and hospital eye services. Further research is needed to evaluate clinical outcomes, cost-effectiveness and the broader therapeutic use of ocular lubricants in acute care.

Key Points

- Independent prescribing activity by optometrists in Wales has substantially increased following National Health Service commissioning, indicating that National Health Service funding acts as a catalyst for professional upskilling and service development.
- Independent prescribing optometrists in Wales are prescribing a wide range of therapeutic agents, particularly for acute inflammatory and infective ocular conditions.
- This study reinforces the importance of developing independent prescribing services within primary eye care.

## Introduction

Around the world, independent prescribing in optometry has been implemented in several countries, including Australia, Canada, New Zealand and the United States [1–4]. In the UK, the 2008 Medicines for Human Use (Prescribing, Miscellaneous Amendments) Order enabled optometrists to become independent prescribers (IP) [5]. In the decade that followed, optometrists in Wales were not given National Health Service (NHS) prescription pads or supported with training or service provision; hence, there were limited numbers of optometrists from Wales taking IP qualifications [6]. This was in contrast to the situation in Scotland, where optometrists were provided with NHS prescription pads and support for training and service provision, and by 2019, approximately 142 community optometrists in Scotland were qualified IPs [7]. This had grown to 600 by 2025 [8].

The optometry prescribing landscape in Wales shifted significantly in March 2020. In response to the COVID-19 pandemic, an Independent Prescribing Optometry Service (IPOS) for acute eye conditions was rapidly commissioned in three of the seven Welsh health boards, enabling IP optometrists to provide NHS-funded acute eye care in primary care settings. This included the issuing of NHS prescriptions for the first time [9]. The model was expanded across Wales in October 2023 with the introduction of the updated NHS Ophthalmic Services Regulations [10, 11], establishing IP-delivered acute care as a national service. Alongside funding for service delivery and access to NHS prescription pads, Health Education and Improvement Wales also supported workforce development by providing funded opportunities for optometrists to gain the Independent Prescribing qualification.

Whilst results from studies of other non-medical prescribers, such as IP nurses and IP pharmacists, give a largely positive view, the evidence base relating to IP optometrists is less robust [12–14]. In 2011, a small survey of the first IP optometrists to qualify in the UK (*n* = 38) reported that feedback from ophthalmologists, general medical practitioners and patients was positive [15]. The same study found that IP optometrist activity had reduced the number of onward referrals to other medical practitioners. A high level of concordance (85%) between hospital IP optometrist and consultant ophthalmologist diagnoses and clinical decision making for acute eye conditions has also been reported [16].

The first study to investigate the effect of independent prescribing by optometrists in a primary care setting (*n* = 278) reported a marked increase (290%) in the number of appointments attributed to the diagnosis and management of anterior segment conditions [7]. More recently, the COVID-19 pandemic resulted in increased IP optometrist activity in the UK. During this period in Scotland, management of acute and urgent eye conditions in the community was found to be safe, effective and impactful on reducing the burden for hospital eye casualty services [17]. In Wales, it was reported that during the pandemic, 78% of patients presenting to an IP optometrist with an acute eye condition were managed within a community setting [9]. The lowest referral rates to other healthcare practitioners and hospital eye services were also recorded in areas that had a commissioned IP pathway.

This study was designed to improve understanding of IP optometry activity from the point at which NHS prescription pads first became available in primary care, and an NHS‑funded IP service was introduced in Wales. We hypothesised that, following the commissioning of NHS IP services, there would be an increase in the number of IP‑qualified optometrists, the number of practices employing them and the volume and range of prescriptions issued using NHS prescription pads. Therefore, the aim of the study was to examine the distribution of IP optometrists and patterns of prescribing for acute eye conditions in Wales between 2020 and 2024, a period during which NHS support for service delivery and training was first introduced and subsequently expanded [9–11].

## Method

NHS Wales Shared Service Partnership (NWSSP) Primary Care Services division provides shared business services to all health boards across Wales. One of their roles is to capture data on the provision of NHS services, including prescribing by primary care contractor professions (including general medical practitioners, pharmacists, dentists and optometrists).

Independent prescribing NHS optometry data extracts began in February 2020, just before the implementation of any commissioned IP optometrist patient pathways in Wales, which commenced in March 2020 [18]. The NWSSP online, open-access repository is accessible for use and re-use under the Open Government Licence (https://nwssp.nhs.wales/ourservices/primary-care-services/general-information/data-and-publications/prescribing-data-extracts/independent-prescribing-optometrist/). Data reported include NHS prescribing by community-based IP optometrists on a monthly basis. Extracts relate to prescriptions only and do not contain any diagnostic information. They also do not detail private prescriptions, prescriptions issued but not dispensed, prescriptions issued in Wales but dispensed outside of Wales or prescriptions issued by IP optometrists working within the Hospital Eye Service.

This study reviewed quantitative prescribing data published by NWSSP from 1st February 2020 to 31st January 2024. Each monthly extract details the following raw data for prescriptions dispensed in Wales: health board code, locality code, optometrist practice code, British National Formulary (BNF) classification code, drug name (proprietary or generic name plus form or strength), number of prescribed items (number of times the medicine was prescribed), drug tariff, cost of drug (net ingredient cost minus NHS discount plus container allowance), quantity prescribed (number of tablets or bottles prescribed) and defined daily dose.

NWSSP data extracts use the BNF classification system to identify prescribed medicines and appliances. Each drug and appliance listed is assigned a unique code which identifies the BNF chapter and subsection listing, and further identifies the chemical substance, product type, strength and formulation where appropriate.

Two BNF chapters primarily denote drugs used in the treatment of acute eye conditions. Topical preparations are detailed in Chapter 11: Eye. This chapter is subdivided into sections for anti-infective preparations, corticosteroids and anti-inflammatory preparations, as well as mydriatics and cycloplegics, glaucoma treatments, local anaesthetics and miscellaneous preparations (which include several ocular lubricants). The remaining ocular lubricants (sodium hyaluronate, sodium chloride eye preparations and some carbomer gels) are listed in Chapter 21: Eye Products. Other chapters which contain medicines that may pertain to acute ocular conditions include Chapter 3 (Section 3.4.1: Antihistamines), Chapter 10 (Section 10.1.1: Non-steroidal anti-inflammatory drugs) and Chapter 13 (skin).

To collate the dataset, IP optometrist monthly extracts were downloaded in comma-separated values (.csv) format. This is a format compatible with visualisation and analysis using Microsoft Excel (Microsoft.com). IP prescription data were collected for each month of the study period with no missing extracts.

To facilitate data analysis, individual monthly datasets were collated into a master spreadsheet housing all raw data over the study period. Once the dataset was complete, it was cleaned prior to analysis. This process involved manually verifying that all entries were complete and verifying BNF classifications to identify any items incorrectly assigned to optometry. To facilitate this, entries were sorted into BNF classification codes. This resulted in the identification of 94 prescriptions either classified as unspecified items (*n* = 67, <0.5% total items prescribed) or incorrectly assigned to optometry (*n* = 27, <0.5% total items prescribed). The criterion to determine this was defined as any medicine listed as ‘Unspecified Drug Code’ or not usually prescribed for conditions affecting the eye and/or the tissues surrounding the eye.

These items belonged to Chapter 1 (Gasto-Intestinal System, *n* = 1), Chapter 2 (Cardiovascular System, *n* = 2), Chapter 4 (Central Nervous System, *n* = 9), Chapter 5 (Infections, *n* = 2), Chapter 6 (Endocrine System, *n* = 2), Chapter 10 (Musculoskeletal and Joint Diseases, *n* = 2), Chapter 12 (Ear, Nose and Oropharynx, *n* = 6), Chapter 19 (Other Drugs and Preparations, *n* = 67), Chapter 21 (Appliances, *n* = 2) and Chapter 22 (Incontinence Appliances, *n* = 1). These items were not included in any subsequent analysis.

Nine-hundred and eighty-four items were prescribed from BNF Chapter 11.6 (Treatment of Glaucoma), of which 920 were topical glaucoma medications and 64 were oral acetazolamide. Whilst some of the topical items may have been prescribed to manage acute conditions, such as steroid response, without patient-level information, it is possible that some of these items may not be attributed to acute eye care. In alignment with this, these preparations have been excluded from Table 3 and identified as a study limitation.

Data analysis was performed using IBM SPSS Statistics for Windows, Version 26 (ibm.com). Descriptive statistics were used to report the distribution and activity of IP optometrists in Wales. Data distribution analysis of active IP optometrist practices across Wales and the number/cost of prescriptions issued in each region was conducted using the Shapiro–Wilk test. Data was found to be normally distributed (*p* = 0.17). After verification that all other assumptions were met, including visual inspection of relationship linearity and extreme outliers, Pearson correlation analysis (*p* = 0.05) was conducted to investigate relationships between these variables.

Cost analysis was performed using the BNF drug tariff for each drug prescribed. BNF proprietary cost information is updated weekly and based on NHS net prices. Albeit this provides a relative cost per unit of medicine, it does not account for Value Added Tax, professional fees and other overheads that may be encountered when generating a prescription. Descriptive statistics were presented to outline regional spending by IP optometrists in Wales, total national spend across the study duration and costs associated with the most widely prescribed medicines for acute eye care by IP optometrists.

## Results

**Fig. 1:**
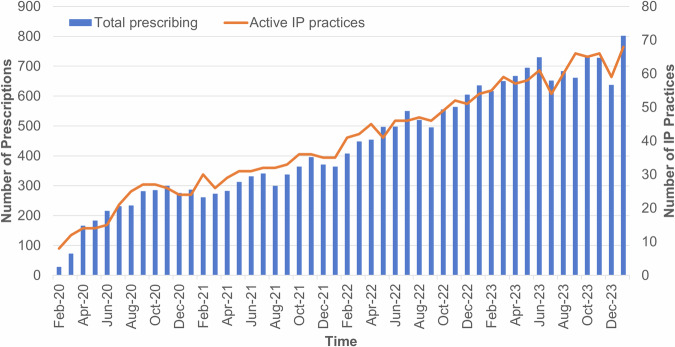
The number of independent prescribing (IP) optometry practices active in Wales and the number of prescriptions issued by IP optometrists from 2020 to 2024. Data for both total prescribing (gradient of trend line = 0.44, *R*^2^ = 0.96) and active IP practices (gradient of trend line = 0.04, *R*^2^ = 0.97) follow a linear increase.

The number of optometry practices with at least 1 active IP optometrist increased from a baseline of eight in February 2020 to 68 in January 2024 (Fig. 1). The number of NHS prescriptions issued by IP optometrists also increased during this time from 2561 in 2020/2021, to 3936, 6230 and 8253 in 2021/2022, 2022/2023 and 2023/2024, respectively, corresponding to an average annual growth of 32.1% per year.

Over the 48-month study period, a total of 20,980 prescriptions were issued by IP optometrists in Wales at a cost of £339,426. A baseline of 28 prescriptions was issued in February 2020; thereafter, the number steadily increased to 802 in January 2024, a growth rate of >2700% across the study period.

### Distribution of IP Optometrist Activity in Wales

Cardiff and Vale University Health Board (UHB), Cwm Taf Morgannwg UHB and Hywel Dda UHB were the first regions to establish commissioned IP services in response to the COVID-19 pandemic. Aneurin Bevan UHB and Powys Teaching Health Board (THB) had IP optometrists in February 2020, but no commissioned IP service. Active IP optometrists eventually emerged in all regions by April 2022.

The distribution of IP active practices did not follow the distribution of population in each region, with some populous areas being underserved by IP optometrists compared to others. The highest distribution of active IP optometry practices was in Cardiff and Vale UHB (Table 1). In contrast, Swansea Bay UHB had the lowest IP optometrist activity, with only one active IP practice during the study period. Although there was overall growth in the number of IP practices over the study period, there were some temporary reductions in activity. This may reflect periods of IP optometrist inactivity (for example, annual leave in Summer and Winter months) or movement of IP optometrists resulting in temporary service disruption.

**Table 1:** Summary of independent prescribing optometry practice activity in Wales.

	Population (*n*)	Practices (*n*)	Practices per 100k (*n*)	Prescriptions (*n*)	Items per Rx (*n*)	Cost (£)
Cardiff and Vale UHB	506k	24	4.74	6234	2.57	115k
Cwm Taf Morgannwg UHB	444k	16	3.60	6708	2.19	120k
Aneurin Bevan UHB	591k	19	3.21	3115	2.25	38k
Powys THB	134k	4	3.01	347	2.19	5k
Hywel Dda UHB	385k	10	2.60	2965	2.30	40k
Betsi Cadwaladr UHB	688k	14	2.03	1539	2.41	20k
Swansea Bay UHB	383k	1	0.26	72	1.29	<1k
**Total/Average**	**3.1M**	**88**	2.81*	**21k**	2.17*	**339k**

Practices estimates correct to January 2024, population estimates correct to mid-year 2022 as per Welsh Government population data available at https://statswales.gov.wales/Catalogue/Population-and-Migration/Population/Estimates/Local-Health-Boards/populationestimates-by-welshhealthboard-year. Prescriptions, items per Rx and cost denote accumulated 2020–2024 values. Average value (as opposed to total) is denoted by *.

*n* number, *THB* Teaching Health Board, *UHB* University Health Board.

After verifying all relevant assumptions were met, including normality testing (Shapiro–Wilk test, *p* = 0.11), positive correlations were found between the number of active IP optometry practices and the number of prescriptions issued (Pearson correlation *r* = 0.81, *p* = 0.001) and spending (Pearson correlation *r* = 0.76, *p* = 0.001) per health board.

### Prescribing Practice for Acute Eye Conditions

A total of 49,162 items (an average of 2.34 items per prescription) were issued by IP optometrists over the study period. Forty-seven percent (*n* = 9861) of the total prescriptions issued by IP optometrists were for generic formulations. A full list of prescribed items is given in Supplement 1. The 30 most readily prescribed items are shown in Table 2.

**Table 2:** Top 30 most readily prescribed items used in presumed acute management by independent prescribing optometrists from 2020 to 2024 and their associated cost.

	Drug name	Concentration	Formulation	Items (*n*)	Spend (£)
1	Chloramphenicol	1.0%	Occ	5073	17,012
2	Pred Forte (Prednisolone, AbbVie Ltd; abbvie.co.uk)	1.0%	Gutt	2721	8014
3	Hylo Forte (Sodium hyaluronate, URSAPHARM Arzneimittel GmbH; ursapharm.de)	0.2%	Gutt	2489	24,985
4	Evolve HA PF (Sodium hyaluronate, Medicom Healthcare Ltd; medicomhealthcare.com)	0.2%	Gutt	2255	13,675
5	Chloramphenicol	0.5%	Gutt	2099	17,470
6	Dexamethasone	0.1%	Gutt	1629	3112
7	Hylo Night PF (URSAPHARM Arzneimittel GmbH; ursapharm.de)	-	Occ	1620	5217
8	Cyclopentolate Hydrochloride	1.0%	Occ	1574	12,543
9	Maxidex (Dexamethasone, Novartis Pharmaceuticals; novartis.com)	0.1%	Gutt	1467	3246
10	FML Ophthalmic (Fluorometholone, AbbVie Ltd; abbvie.com)	0.1%	Gutt	1436	2649
11	Xailin Night PF (Medicom Healthcare Ltd; medicomhealthcare.com)	-	Occ	1309	3756
12	Hylo-Tear (Sodium hyaluronate, URSAPHARM Arzneimittel GmbH; ursapharm.de)	0.1%	Gutt	1241	10,290
13	Ganciclovir	0.15%	Gutt	1116	22,821
14	Prednisolone Acetate	1.0%	Gutt	1016	3155
15	Maxitrol (Novartis Pharmaceuticals; novartis.com)	0.1%	Occ	943	1416
16	Mydrilate (Cyclopentolate hydrochloride, Esteve Pharmaceuticals Ltd; esteve.com)	1.0%	Gutt	920	7037
17	Xailin Eye Gel (Medicom Healthcare Ltd; medicomhealthcare.com)	0.2%	Occ	792	3139
18	Maxitrol (Novartis Pharmaceuticals; novartis.com)	0.1%	Gutt	747	1252
19	Aciclovir	400 mg	PO	719	2499
20	Olopatadine	0.1%	Gutt	697	4059
21	Carbomer 980	0.2%	Gutt	673	2531
22	Opatanol (Olopatadine, Novartis Pharmaceuticals; novartis.com)	0.1%	Gutt	636	3249
23	Co-Amoxiclav	500/125 mg	PO	615	2244
24	Viscotears (Carbomer, Bausch and Lomb Inc; bausch.com)	0.2%	Occ	560	948
25	Flurone (Flurometholone, Biopharma Ltd; biopharmaltd.com)	0.1%	Gutt	542	1226
26	Medicom Carbomer (Medicom Healthcare Ltd; medicom.com)	0.2%	Occ	534	2010
27	Ciloxan (Ciprofloxacin Hydrochloride, Novartis Pharmaceuticals; novartis.com)	0.3%	Gutt	532	2878
28	Softacort PF (Hydrocortisone, Théa Pharmaceuticals Ltd; thea-pharmaceuticals.co.uk)	0.3%	Gutt	493	9024
29	Virgan (Ganciclovir, Théa Pharmaceuticals Ltd; thea-pharmaceuticals.co.uk)	0.15%	Gutt	492	10,629
30	Sodium Cromoglycate	2.0%	Gutt	449	3049

*Gutt* drops, *Occ* ointment, *PF* preservative-free, *PO* oral.

The five most frequently prescribed individual items (*n* = number of items prescribed, % all items prescribed by IP optometrists) were (i) Chloramphenicol 1% eye ointment (*n* = 5073, 10.3%), (ii) Pred Forte 1% eye drops (*n* = 2721, 5.5%, AbbVie Ltd; abbvie.co.uk), (iii) Hylo Forte sodium hyaluronate eye drops (*n* = 2489, 5.1%, URSAPHARM Arzneimittel GmbH; ursapharm.de), Evolve HA sodium hyaluronate 0.2% eye drops 10 ml preservative-free (PF) (*n* = 2255, 4.6%, Medicom Healthcare Ltd; medicomhealthcare.com) and (v) Chloramphenicol eye drops 0.5% (*n* = 2099, 4.3%).

The number of prescriptions issued monthly in the most widely prescribed BNF chapter (11: Eye) increased by 2200% (*n* = 22 in February 2020 to *n* = 518 in January 2024). The average yearly increase (±SD) was 1198 prescriptions (±239) dispensed for drugs listed within this chapter. There were no obvious seasonal variations. Table 3 provides an overview of prescriptions from each section of BNF Chapter 11. The largest number of prescriptions was issued from the following drug classifications: Corticosteroids and other anti-inflammatory preparations (*n* = 5777), anti-infective eye preparations (*n* = 4374), miscellaneous ophthalmic preparations (*n* = 1633) and mydriatics and cycloplegics (*n* = 1120).

**Table 3:** Quantity of prescriptions and number of items issued from the British National Formulary Chapter 11: Eye.

Section	Title/Subsection	Prescriptions (*n*)	Items (*n*)	Items per Rx (*n*)
11.3	**Anti-infective eye preparations**	**4374**	**11,176**	**2.56**
11.3.1	Antibacterials	3572	9515	2.66
11.3.2	Antifungals	0	0	0.00
11.3.3	Antivirals	802	1661	2.07
11.4	**Anti-inflammatory preparations**	**5777**	**14,405**	**2.49**
11.4.1	Corticosteroids	4507	11,903	2.64
11.4.2	Other anti-inflammatory preparations	1270	2502	1.97
11.5	**Mydriatics and cycloplegics**	**1120**	**2667**	**2.38**
11.7	**Local anaesthetics**	**0**	**0**	**0.00**
11.8	**Miscellaneous ophthalmic preparations**	**1633**	**2997**	**1.84**
11.8.1	Tear deficiency, eye lubricant and astringent	1225	2425	1.98
11.8.2	Ocular diagnostic and peri-operative prep	408	572	1.40
11.8.3	Other eye preparations	0	0	0.00

Section titles in bold to differentiate from subsections. Drugs from section 11.6: Treatment of Glaucoma have been excluded as most were unlikely to be associated with acute eye care.

*n* number, *Rx* prescription.

IP optometrists were found to prescribe corticosteroids more readily than any other anti-inflammatory preparation. The most widely prescribed anti-infective eye preparations were antibacterials, followed by antivirals. No antifungal or local anaesthetic prescriptions were issued. The most readily prescribed drugs from within the miscellaneous subcategory were preparations for tear deficiency, eye lubricants and astringents.

Comparatively, prescriptions issued from other BNF chapters were far fewer, accounting for 33.8% of all IP optometrist prescriptions. The second most widely prescribed chapter was Chapter 21: Appliances. This is a pseudo-BNF chapter that contains a subsection (21.30) reserved for eye preparations not included elsewhere in the BNF, e.g., some additional ocular lubricants and astringents. A total of 4719 prescriptions were issued from chapter 21 over the study period, accounting for 13,003 items. The majority of prescriptions were issued from subsections relating to eye products (treatment of dry eye disease) and emollients.

Other drug classifications prescribed by optometrists included oral medications listed in Chapter 5 (Infections). A total of 1175 oral antibacterial prescriptions were issued, of which penicillins (*n* = 732) and tetracyclines (*n* = 267) were most prevalent. Medicines prescribed in lower numbers included oral anti-viral drugs (*n* = 522; Chapter 5), medicines for skin conditions (*n* = 345; Chapter 13) and oral antihistamines (*n* = 273; Chapter 3).

### Cost Analysis

**Fig. 2:**
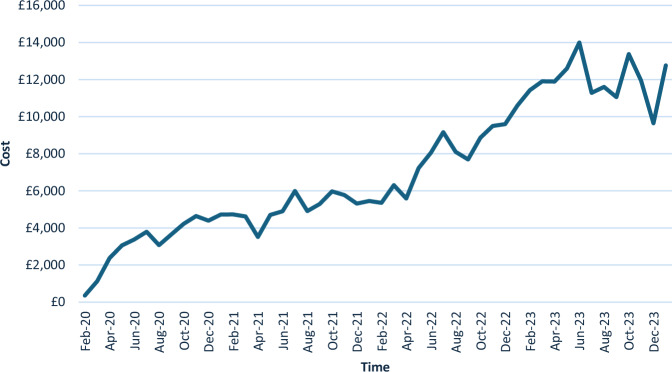
Monthly spending on prescriptions issued by independent prescribing optometrists in Wales from 2020 to 2024. The data follow a linear relationship (gradient of trend line = 7.91, *R*^2^ = 0.90).

NHS Prescriptions from IP optometrists cost £339,426 over the study period. Spending on prescriptions increased steadily each month (Fig. 2) with an overall increase of 3500% in 4 years (£352 in February 2020 to £12,700 in January 2024). The average yearly increase (±SD) was £34,902 (±12,536). At a local level, spending correlated with the duration of IP activity in each health board, with the highest spending in health boards that commissioned services earliest in response to the COVID pandemic.

The top 30 drugs per expenditure by IP optometrists are given in Table 4. The average cost of a prescription item issued by IP optometry was £6.90, and the amount spent (£142,772) on generic medications accounted for 42.1% of all costs. The highest spending individual items prescribed by IP optometrists over the study period were (i) Hylo Forte sodium hyaluronate 0.2% eye drops (£24,985), Ganciclovir 0.15% eye gel (£22,821), Chloramphenicol 0.5% eye drops preserved (£17,469), Chloramphenicol 1% eye ointment (£17,011) and Ikervis (ciclosporin) 0.1% PF eye drops (£14,925).

**Table 4:** Independent prescribing optometrists’ top 30 drug expenditure from 2020 to 2024 and the associated number of items prescribed.

	Drug name	Concentration	Formulation	Spend (£)	Items (*n*)
1	Hylo Forte (Sodium hyaluronate, URSAPHARM Arzneimittel GmbH; ursapharm.de)	0.2%	Gutt	24,985	2489
2	Ganciclovir	0.15%	Gutt	22,821	1116
3	Chloramphenicol	0.5%	Gutt	17,470	2099
4	Chloramphenicol	1.0%	Occ	17,012	5073
5	Ikervis PF (Santen Pharmaceutical Ltd; santen.com)	0.1%	Gutt	14,925	143
6	Evolve HA PF (Sodium hyaluronate, Medicom Healthcare Ltd; medicomhealthcare.com)	0.2%	Gutt	13,675	2255
7	Cyclopentolate Hydrochloride	1.0%	Occ	12,543	1574
8	Virgan (Ganciclovir, Théa Pharmaceuticals Ltd; thea-pharmaceuticals.co.uk)	0.15%	Gutt	10,629	492
9	Hylo-Tear (Sodium hyaluronate, URSAPHARM Arzneimittel GmbH; ursapharm.de)	0.1%	Gutt	10,290	1241
10	Softacort PF (Hydrocortisone, Théa Pharmaceuticals Ltd; thea-pharmaceuticals.co.uk)	0.3%	Gutt	9024	493
11	Pred Forte (Prednisolone, AbbVie Ltd; abbvie.co.uk)	1.0%	Gutt	8014	2721
12	Mydrilate (Cyclopentolate hydrochloride, Esteve Pharmaceuticals Ltd; esteve.com)	1.0%	Gutt	7037	920
13	Dexafree PF (Dexamethasone, Théa Pharmaceuticals Ltd; thea-pharmaceuticals.co.uk)	0.1%	Gutt	5640	363
14	iLube (Acetylcysteine, Rayner Pharmaceuticals Ltd; rayner.com)	5.0%	Gutt	5400	101
15	Dexamethasone PF	0.1%	Gutt	5257	167
16	Hylo Night PF (URSAPHARM Arzneimittel GmbH; ursapharm.de)	-	Occ	5217	1620
17	Dropodex PF (Dexamethasone, Rayner Pharmaceuticals Ltd; rayner.com)	0.1%	Gutt	4516	193
18	Thealoz Duo (Sodium hyaluronate, Théa Pharmaceuticals Ltd; thea-pharmaceuticals.co.uk)	0.15%	Gutt	4284	413
19	Atropine	1.0%	Gutt	4109	23
20	Olopatadine	0.1%	Gutt	4059	697
21	Xailin Night PF (Medicom Healthcare Ltd; medicomhealthcare.com)	-	Occ	3756	1309
22	Sodium Chloride	5.0%	Gutt	3503	115
23	Fusidic Acid	1.0%	Gutt	3484	111
24	Opatanol (Olopatadine, Novartis Pharmaceuticals; novartis.com)	0.1%	Gutt	3249	636
25	Maxidex (Dexamethasone, Novartis Pharmaceuticals; novartis.com)	0.1%	Gutt	3246	1467
26	Prednisolone Acetate	1.0%	Gutt	3155	1016
27	Xailin Eye Gel (Medicom Healthcare Ltd; medicomhealthcare.com)	0.2%	Occ	3139	792
28	Dexamethasone	0.1%	Gutt	3112	1629
29	Sodium Cromoglycate	2.0%	Gutt	3049	449
30	Ciloxan (Ciprofloxacin hydrochloride, Novartis Pharmaceuticals; novartis.com)	0.3%	Gutt	2878	532

*Gutt* drops, *Occ* ointment, *PF* preservative-free, *PO* oral.

A total of 5923 prescriptions (15,400 items, 31.3% of all items prescribed) were issued for ocular lubrication products over the 48-month period. This attributed to 34.0% of all IP optometrist spending. The total cost was £115,500 (average 2.6 items issued per prescription), making this group the most frequently prescribed items and the highest spend by IP optometrists. The second highest spending by IP optometry was for anti-infective eye preparations. A total of £97,646 was spent from this classification, from which a large proportion can be attributed to the provision of all formulations of chloramphenicol (£38,083) and ganciclovir (£33,451).

## Discussion

This study represents a quantitative analysis of prescribing practices among primary care IP optometrists in Wales, contributing novel evidence to the limited body of research on IP optometry within the UK.

In line with other studies assessing the impact of non-medical prescribing, this study found a trend of increasing optometry prescribing activity [19, 20]. Overall prescribing activity increased each year of the study, and within each health board. The limited level of IP activity in primary care optometry prior to the commissioning of these services indicates that the availability of NHS funding and prescribing rights acts as a catalyst for professional upskilling and service development. Similar trends have been reported in Scotland [7].

There was an inconsistency in IP optometry activity across the health boards. Cardiff University UHB, Cwm Taff UHB and Aneurin Bevan UHB were better served by the end of the study period. The full reasons remain unclear. It could be that there is variation in optometry practice distribution in Wales. However, the early commissioning of the IPOS service prior to the implementation of the updated NHS Ophthalmic Services Regulations in Wales [10, 11] and the establishment of the first ‘Teach and Treat’ clinic in Cardiff University, providing placements for those wishing to do the IP qualification in these areas, may have contributed to the faster uptake. Comparatively, health boards with the longest history of NHS IP services were the most active throughout the study period. Teach and Treat Clinics and ‘Advanced Training Practices’ to support the training of IP optometrists were established in North and South-West Wales in 2024, and it will be important to determine if this targeted strategy has reduced regional disparities in service uptake to ensure equitable access across Wales.

In 2018, prior to the study period, Accident and Emergency departments, general medical practitioners (GPs), community pharmacists and optometrists collectively managed 365,044 episodes of acute eye care in Wales, of which 51.8% were managed by optometrists [21]. Notably, 168,877 episodes were managed within general practice. Considering the increasing demand and workload pressures in general practice, transferring some responsibilities from GPs to optometrists could help free up general practice capacity. In Scotland, as independent prescribing increased, workload in general practice in terms of the burden of acute eye care decreased [20]. Further research is needed to assess whether the increase in activity by IP optometrists in primary care in Wales has helped to reduce the burden of acute eye care on GPs.

The top 10 most commonly prescribed medications by IP optometrists reflected heavy use of anti-microbial, anti-inflammatory and ocular lubricant items. This correlates with GP prescribing activity for acute eye disease in Wales [21]. The widespread use of antibiotic preparations aligns with the need to provide broad-spectrum cover against infection for a range of commonly encountered ocular conditions. Albeit rare in the general population (15–52 per 100k), the most prevalent form of uveitis is anterior uveitis (75% of all cases) [22]. This incidence, combined with the recurrent nature of the condition and its potential persistence after cataract surgery, may explain the frequent prescription of corticosteroid eye drops. Ocular lubricants are in widespread usage and can be issued for both acute (e.g., viral conjunctivitis) and chronic (e.g., recurrent corneal erosions) conditions, but are frequently prescribed for dry eye disease, which is a chronic disorder that often presents with acute symptoms [23].

The growth of IP pharmacy has run parallel to IP optometry in Wales. In 2016, the number of IP pharmacists in Wales was 167; this grew to 483 in 2020 [24]. The Welsh Government Future of Community Pharmacy in Wales Strategy [25] outlined that from April 2022, any pharmacy would be enabled to provide a new national IP pharmacy service, subject to employing a suitably qualified and competent IP pharmacist. A recent study exploring the experience of IP pharmacists involved in the pilot Pharmacy Independent Prescriber Service (PIPS) reported high levels of satisfaction and positive attitude towards the service from pharmacists and commissioners [24]. Given that PIPS and IPOS are recent initiatives, it is not possible at this stage to predict how each service will impact the other or GP activity in Wales in the future. However, given the growing body of evidence relating to enhanced patient experience with prescribing services in the community and the Welsh Government’s long-term plan for health and social care [26], continued investment in this sector is anticipated.

At the time of writing, a UK-wide government consultation is in progress to consider the expansion of the range of medicines that optometrists and contact lens opticians (CLOs) may supply for the management of minor eye conditions, including allergic and bacterial conjunctivitis [27]. The proposed amendments seek to enhance the therapeutic scope of these practitioners, enabling them to manage a broader spectrum of minor ocular disorders. During the 4-year study period of this analysis, IP optometrists in primary care demonstrated a considerably wider prescribing range than that envisaged by the proposed legislation. IP optometrists frequently prescribed topical corticosteroids for inflammatory conditions such as anterior uveitis, anti-viral agents, including ganciclovir eye gel for herpes simplex keratitis and oral antibiotics for infections such as pre-septal cellulitis. Accordingly, while the proposed legislative expansion represents a positive development for the optometric profession, it is unlikely to obviate the ongoing need for IP optometrists within primary care.

Generic prescribing is acknowledged as desirable and representative of high-quality prescribing in Wales and the UK, as it can reduce errors, facilitate quicker supply and provide cost savings for the NHS [28, 29]. In England, 81% of primary care prescription items were prescribed generically [30]. In this study, generic formulations accounted for 47.0% of all prescribing by IP optometrists in Wales. The preference to prescribe branded alternatives is out of line with other NHS prescribers and indicates an area in which additional education may be helpful for optometry professionals who are new to NHS prescribing.

Ocular lubricants were among the most frequently prescribed medications, with three of the 10 most prescribed agents by IP optometrists falling within this category. These preparations are primarily used for the management of dry eye disease, including severe cases that may result in visual disturbance and substantial ocular discomfort. However, within independent prescribing contexts, ocular lubricants may also be employed to support corneal healing and enhance patient comfort following ocular trauma, as well as in the management of other conditions such as viral and allergic conjunctivitis. Despite the finding that they are frequently used in an acute eye care setting, there remains limited empirical evidence regarding these broader clinical applications, highlighting an area that warrants further investigation.

### Limitations of the Study

This study relied on the accuracy of data extracts provided by the NWSSP. Some data cleaning was required to remove items incorrectly attributed to IP optometry or unrelated to ocular conditions. Although some excluded items may have represented genuine IP acute eye care clinic activity, the small volume of removals (*n* = 94, 0.45% of total prescriptions) is unlikely to have materially affected the findings. Conversely, all glaucoma prescriptions (*n* = 984 over the study period, 4.7% of total prescriptions) were included in the monthly analyses; it is possible that some of these may not be attributed to acute eye care.

As the NWSSP only collates data relating to the provision of NHS services, data associated with private prescriptions and over-the-counter sales were missing from the analyses. In addition, as optometrist prescribing data was investigated, medications supplied by other healthcare professionals, e.g., via the Common Ailments Service, were not captured. As the dataset was specific to prescribing practice in Wales, further data loss may have occurred when prescriptions were dispensed outside Wales, particularly in the border regions. However, this effect is expected to be minimal.

As the data collected did not specify individual optometrist output, it was not possible to qualify the exact number of IP optometrists within each region. This may have had an effect on the data, i.e., a large increase in IP optometrists could be hidden by a small increase in active practices and vice versa. The latest workforce figures, published in 2021, reported 45 qualified IP optometrists in Wales (6% of the workforce) [31]. Following a data request, unpublished workforce data from health boards reported 93 IP optometrists active across both primary and secondary care services by the end of the study period.

The absence of diagnostic or patient-level information also limits interpretation, as it was not possible to assess indications for treatment, patient outcomes or adherence to clinical management guidelines. Consequently, the study reflects prescribing activity rather than clinical effectiveness or quality of care.

### Further Research

Further research is required to evaluate the broader impact and clinical effectiveness of IP within optometric practice. Studies should investigate whether increased prescribing activity among IP optometrists translates to measurable reductions in workload across general practice and emergency eye care services. Economic evaluations would also be valuable to assess the cost-effectiveness of expanding IP services and their contribution to NHS resource optimisation.

Research about patient knowledge, understanding and perceptions of IP optometry services may help progress public awareness campaigns and aid in examining barriers to service uptake in underserved regions to inform strategies for equitable service delivery.

Given the high frequency of ocular lubricant prescribing, further investigation into their broader therapeutic applications in managing acute eye conditions could provide valuable evidence to support best practice. In addition, the range of prescribing suggests optometrists are treating a number of complex ocular conditions. Further investigation with patient-level data could support further development of clinical management guidelines.

Finally, as UK policy continues to evolve regarding the range of medicines that optometrists and CLOs may supply, research should evaluate the impact of these legislative changes on clinical practice and the continuing need for IP optometrists within primary care.

## Conclusion

This study provides the first comprehensive analysis of independent prescribing activity by optometrists in Wales, demonstrating a substantial increase in service provision following NHS commissioning. The data revealed that IP optometrists are prescribing a wide range of therapeutic agents, particularly for acute inflammatory and infective ocular conditions and are increasingly relied upon to deliver frontline eye care. The high frequency and cost of ocular lubricant prescribing suggest their central role in acute eye care, though further research is needed to clarify their broader clinical applications.

As policy discussions continue regarding the expansion of optometric prescribing rights, this study reinforces the importance of maintaining and developing IP services within primary care. Future research should focus on patient outcomes, service impact on other healthcare providers and the development of clinical guidelines to support safe and effective prescribing by optometrists.

## Supplementary Information

Supplement 1

## Data Availability

The datasets used and analysed for the present study are available from the corresponding author upon reasonable request.
