# Supplementary material for: The Rise of Independent Prescribing by Optometrists in Wales 2020–2024: Number of Practices, Drugs and Costs
Source: Ophthalmic Physiol Opt. 2026 May 4;46(3):518–26. doi: 10.1007/s44402-026-00097-1 (PMC13369029; doi:10.1007/s44402-026-00097-1)
Supplement: Supplementary file 1 — Supplement 1 [file 44402_2026_97_MOESM1_ESM.docx]

| **Drug Name** | **Spending (£)** | **Items (n)** |
| --- | --- | --- |
| Chloramphenicol 1% eye ointment | £17011.74 | 5073 |
| Pred Forte Eye Drops 1% (AbbVie Ltd; abbvie.co.uk) | £8014.26 | 2721 |
| Hylo-Forte Sod Hyaluronate Eye Drops (URSAPHARM Arzneimittel GmbH; ursapharm.de) | £24985.14 | 2489 |
| Evolve HA Sod Hyaluronate 0.2% 10ml PF (Medicom Healthcare Ltd; medicomhealthcare.com) | £13674.64 | 2255 |
| Chloramphenicol Eye Drops 0.5% | £17469.72 | 2099 |
| Dexamethasone Eye Drops 0.1% | £3111.92 | 1629 |
| Hylo Night eye ointment PF (URSAPHARM Arzneimittel GmbH; ursapharm.de) | £5217.36 | 1620 |
| Cyclopentolate Hydrochloride Eye Drops 1% | £12543.07 | 1574 |
| Maxidex Eye Drops (Novartis Pharmaceuticals Ltd; novartis.com) | £3245.87 | 1467 |
| FML Ophthalmic Suspension 0.1% (AbbVie Ltd; abbvie.co.uk) | £2648.83 | 1436 |
| Xailin Night Paraf Eye Ointment P/F 5g (VISUfarma Ltd; visufarma.com) | £3755.85 | 1309 |
| Hylo Sodium Hyaluronate Eye Drops (URSAPHARM Arzneimittel GmbH; ursapharm.de) | £10289.64 | 1241 |
| Ganciclovir Eye Gel 0.15% (Thea Pharmaceuticals Ltd; thea-pharmaceuticals.co.uk) | £22821.46 | 1116 |
| Prednisolone Acetate Eye Drops 1% | £3154.82 | 1016 |
| Maxitrol Eye Ointment (Novartis Pharmaceuticals Ltd; novartis.com) | £1415.73 | 943 |
| Mydrilate 1% (Esteve Pharmaceuticals Ltd; esteve.com) | £7037.09 | 920 |
| Xailin Carbomer Eye Gel 10g (VISUfarma Ltd; visufarma.com) | £3139.33 | 792 |
| Maxitrol Eye Drops (Novartis Pharmaceuticals Ltd; novartis.com) | £1251.84 | 747 |
| Aciclovir Tablet 400mg | £2499.05 | 719 |
| Olopatadine Eye Drops 1mg/ml | £4058.96 | 697 |
| Carbomer 980 Gel Eye Drops 0.2% | £2531.07 | 673 |
| Opatanol_Eye Drops 1mg/ml (Novartis Pharmaceuticals Ltd; novartis.com) | £3248.66 | 636 |
| Co-amoxiclav 500mg/125mg tablets | £2243.83 | 615 |
| Viscotears Liquid Gel 0.2% (Bausch and Lomb; bausch.com) | £947.94 | 560 |
| Flurone (Flurometholone, Biopharma Ltd; biopharmaltd.com) | £1225.87 | 542 |
| Medicom Carbomer 0.2% Eye Gel 10g (Medicom Healthcare Ltd; medicomhealthcare.com) | £2009.91 | 534 |
| Ciloxan Eye Drops 0.3% (Novartis Pharmaceuticals Ltd; novartis.com) | £2877.58 | 532 |
| Softacort Eye Drops 3.35mg/ml 0.4ml Ud (Thea Pharmaceuticals Ltd; thea-pharmaceuticals.co.uk) | £9024.21 | 493 |
| Virgan Eye Gel 0.15% (Thea Pharmaceuticals Ltd; thea-pharmaceuticals.co.uk) | £10629.36 | 492 |
| Sodium Cromoglicate Eye Drops 2% | £3048.67 | 449 |
| Thealoz Duo Eye Drops 10ml PF (Thea Pharmaceuticals Ltd; thea-pharmaceuticals.co.uk) | £4283.80 | 413 |
| Acular Ophthalmic Solution 0.5% (AbbVie Ltd; abbvie.co.uk) | £1534.61 | 411 |
| Dexafree Eye Drops 1mg/ml 0.4ml Ud (Thea Pharmaceuticals Ltd; thea-pharmaceuticals.co.uk) | £5640.04 | 363 |
| Hypromellose 0.3% eye drops | £515.47 | 345 |
| Olopatadine 1mg/ml eye drops | £1358.49 | 288 |
| Celluvisc Eye Drops 1% 0.4ml Ud (AbbVie Ltd; abbvie.co.uk) | £2020.66 | 261 |
| Ciprofloxacin Hydrochloride Eye Drops 0.3% | £1186.50 | 247 |
| Levofloxacin 5mg/ml eye drops | £1938.24 | 233 |
| Doxycycline 100mg capsules | £1532.51 | 224 |
| Co-amoxiclav 250mg/125mg tablets | £362.19 | 217 |
| Dropodex Eye Drops 0.1% 0.4ml Ud (Rayner Pharmaceuticals Limited; rayner.com) | £4515.81 | 193 |
| Aciclovir Tablet 800mg | £726.62 | 189 |
| Latanoprost Eye Drops 50mcg/ml | £627.71 | 188 |
| Xailin HA 0.2% Eye Drops 10ml PF (VISUfarma Ltd; visufarma.com) | £1478.70 | 187 |
| Ketofall Eye Drops 0.25mg/ml 0.4ml Ud (Scope Ophthalmics Ltd; scopeeyecare.com) | £2147.71 | 183 |
| Dexamethasone Eye Drops 0.1% 0.4ml Ud PF | £5256.96 | 167 |
| Blink Intensive Tears Sod Hyaluronate Eye Drops (Abbott Medical Optics Ltd; abott.co.uk) | £485.77 | 153 |
| Minims Chloramphenicol Eye Drops 0.5% Ud PF | £2193.10 | 150 |
| Celluvisc Eye Drops 0.5% 0.4ml Ud (AbbVie Ltd; abbvie.co.uk) | £1683.86 | 146 |
| Flucloxacillin Sodium Capsule 500mg | £384.65 | 144 |
| Ikervis Eye Drops 0.1% 0.3ml Ud (Santen UK Limited; santen.uk) | £14924.90 | 143 |
| Azyter Eye Drops 15mg/g 0.25 Ud PF (Thea Pharmaceuticals Ltd; thea-pharmaceuticals.co.uk) | £2422.06 | 139 |
| Prednisolone Sodium Phosphate Ear/eye Drops 0.5% | £362.80 | 139 |
| Blumont Hypromellose 0.3% Eye Drops 10ml (Blumont Healthcare Ltd; blumonthealthcare.com) | £202.91 | 135 |
| Hydrocortisone 1% cream | £220.11 | 134 |
| Dexamethasone Eye Drops 0.1% PF | £1570.48 | 133 |
| Fexofenadine 120mg tablets | £225.95 | 129 |
| Ofloxacin Eye Drops 0.3% | £304.60 | 127 |
| Brinzolamide 10mg/ml eye drops | £567.05 | 118 |
| Liquifilm 1.4% Polyvinyl Alcohol Eye Drops 15ml (AbbVie Ltd; abbvie.co.uk) | £233.15 | 116 |
| Sodium Chloride 5% Eye Drops | £3502.61 | 115 |
| Fusidic acid 1% modified-release eye drops | £3483.83 | 111 |
| Lumecare Evolve Carmellose 0.5% 10ml PF (Medicom Healthcare Ltd; medicomhealthcare.com) | £564.18 | 108 |
| Ilube Eye Drops 5% (Rayner Pharmaceuticals Ltd; Rayner.com) | £5399.62 | 101 |
| Minims Dexamethasone 0.1% Ud PF | £1894.45 | 97 |
| Flucloxacillin 250mg capsules | £181.46 | 96 |
| Monopost Eye Drops 50mcg/ml 0.2mlUd PF (Théa Pharmaceuticals Ltd; thea-pharmaceuticals.co.uk) | £1595.79 | 93 |
| Minims Prednisolone Sodium Phosphate Ud 0.5% | £1526.98 | 93 |
| AaqEye HA 0.2% eye drops (Essential-Healthcare Ltd; essential-healthcare.co.uk) | £381.67 | 92 |
| Evolve Carbomer 980 Eye Gel 10g (Medicom Healthcare Ltd; medicomhealthcare.com) | £307.53 | 90 |
| Clinitas Carbomer Eye Gel (Altacor Ltd; altacorpharma.co.uk) | £154.77 | 86 |
| Vita-POS Parafin Eye Ointment (Ursapharm Arzneimittel GmbH; ursapharm.de) | £263.07 | 85 |
| Exocin Ophthalmic Solution 0.3% (AbbVie Ltd; abbvie.co.uk) | £190.96 | 85 |
| Amoxicillin 500mg capsules | £148.75 | 79 |
| Aciclovir Tablet 200mg | £229.48 | 77 |
| Doxycycline 50mg capsules | £388.92 | 76 |
| Aactive hyaluronic acid 0.2% eye drops (TriOn Pharma Ltd; trionpharma.co.uk) | £270.83 | 71 |
| Ketorolac Trometamol Eye Drops 0.5% | £554.70 | 70 |
| Erythromycin 250mg gastro-resistant tablets | £237.32 | 69 |
| Xalatan Eye Drops 50mcg/ml (Pfizer; pfizer.co.uk) | £928.50 | 68 |
| Desloratadine 5mg tablets | £209.33 | 67 |
| Loratadine 10mg tablets | £55.36 | 66 |
| Optive Fusion Sodium Hyaluronate Eye Drops 10ml (AbbVie Ltd; abbvie.co.uk) | £486.29 | 65 |
| Azithromycin 500mg tablets | £99.26 | 65 |
| Hydrocortisone 1% ointment | £149.43 | 61 |
| Cetirizine Hydrochloride Tablet 10mg | £45.47 | 60 |
| Hylo-Care Sodium Hyaluronate Eye Drops (Ursapharm Arzneimittel GmbH; ursapharm.de) | £622.66 | 58 |
| Alissa 0.3% Hypromellose Eye Drops 10ml PF (Alissa Healthcare Research Ltd; alissahealthcare.com) | £353.83 | 58 |
| Dexamethasone Eye Drops 0.1% Ud | £1916.16 | 57 |
| Cosopt Ocumeter Plus Eye Drops (Merck and Co Inc; merck.com) | £664.68 | 57 |
| Acetazolamide 250mg tablets | £74.92 | 56 |
| Mydriacyl Eye Drops 1% (Alcon Eye Care UK Limited; alcon.com) | £82.43 | 55 |
| Lymecycline 408mg capsules | £472.27 | 54 |
| Aciclovir 3% eye ointment | £2389.05 | 53 |
| Lumecare Hypromellose Eye Drops (Medicom Healthcare Ltd; medicomhealthcare.com) | £94.44 | 53 |
| Chloramphenicol Eye Drops 0.5% Ud | £1011.98 | 50 |
| Ketotifen Fumarate Eye Drops 250mcg/ml | £442.92 | 49 |
| AacuLose Hypromellose 0.3% eye drops (TriOn Pharma Ltd; trionpharma.co.uk) | £32.33 | 48 |
| Lumigan Eye Drops 100mcg/ml (AbbVie Ltd; abbvie.co.uk) | £804.65 | 47 |
| Clinitas Multi 0.4% Eye Drops 10ml PF (Altacor Ltd; altacorpharma.co.uk) | £521.38 | 46 |
| Viscotears Liquid Gel 0.2% Ud (Bausch and Lomb; bausch.com) | £400.60 | 46 |
| Azarga Eye Drops (Novartis Pharmaceuticals Ltd; novartis.com) | £585.97 | 45 |
| Ketotifen Fumarate Eye Drops 250mcg/ml 0.4ml Ud | £768.74 | 44 |
| Eythalm Eye Drops 1mg/ml (Aspire Pharma Ltd; aspirepharma.com) | £408.77 | 44 |
| SodiEye 5% Soiumd Chloride Eye Dps 10ml PF (TriOn Pharma Ltd; trionpharma.co.uk) | £804.46 | 43 |
| Timolol Maleate Eye Drops 0.25% | £103.59 | 41 |
| Soolantra Cream 10mg/g (Galderma Ltd; galderma.com) | £1022.56 | 40 |
| Ganfort Eye Drops (AbbVie Ltd; abbvie.co.uk) | £750.82 | 39 |
| Azopt Eye Drops 10mg/ml (Alcon Eye Care UK Limited; alcon.com) | £296.29 | 39 |
| Systane Balance Hydroxypropyl Eye Drops (Alcon Eye Care UK Limited; alcon.com) | £294.28 | 39 |
| VisuXL Gel eye drops PF (VISUfarma Ltd; visufarma.com) | £285.01 | 39 |
| Cosopt Eye Drops 0.2ml Ud PF (Santen UK Limited; santen.uk) | £1198.27 | 38 |
| Lodoxamide Trometamol Eye Drops 0.1% (Novartis Pharmaceuticals Ltd; novartis.com) | £218.78 | 38 |
| Cyclopentolate Hydrochloride Eye Drops 0.5% | £323.58 | 37 |
| Otrivine-Antistin Eye Drops (Thea Pharmaceuticals Ltd; thea-pharmaceuticals.co.uk) | £115.76 | 37 |
| Simbrinza Suspension Eye Drops 10mg/ml / 2mg/ml (Novartis Pharmaceuticals Ltd; novartis.com) | £403.68 | 35 |
| Chloramphenicol Eye Drops 0.5% PF | £377.12 | 35 |
| VisuXL Sodium Hyaluronate Eye Drops 10ml PF (VISUfarma Ltd; visufarma.com) | £421.73 | 34 |
| Carbomer 980 Gel Eye Drops 0.2% Ud | £374.70 | 34 |
| Levofloxacin Eye Drops 5mg/ml 0.3ml Ud PF | £595.60 | 33 |
| Tobradex Eye Drops (Novartis Pharmaceuticals Ltd; novartis.com) | £219.81 | 33 |
| Teardew 0.5% Hypromellose Eye Drops 10ml (Sai-Meds Ltd; sai-med.com) | £36.36 | 32 |
| Azithromycin Eye Drops 15mg/g 0.25g Ud PF | £325.41 | 31 |
| Hy-Opti 0.1% Sodium Hyaluronate Eye Drops 10ml PF (Alissa Healthcare Research Ltd; alissahealthcare.com) | £179.41 | 31 |
| Lumigan Eye Drops 300mcg/ml 0.4ml Ud (AbbVie Ltd; abbvie.co.uk) | £930.39 | 29 |
| Oftaquix Eye Drops 5mg/ml 0.3ml Ud (Santen UK Limited; santen.uk) | £572.43 | 29 |
| Medicom Carmellose 0.5% Eye Drops 10ml (Medicom Healthcare Ltd; medicomhealthcare.com) | £251.47 | 29 |
| Sodium Cromoglicate Eye Drops Aqueous 2% PF | £164.83 | 29 |
| Betnesol Ear/eye/nose Drops 0.1% (RPH Pharmaceuticals AB; recipharm.com) | £62.91 | 29 |
| Flurbiprofen 100mg tablets | £985.55 | 28 |
| Clinitas Sodium Hyaluronate Eye Drops 0.5ml Ud (Altacor Ltd; altacorpharma.co.uk) | £531.06 | 28 |
| Brimonidine Tartrate Eye Drops 0.2% (AbbVie Ltd; abbvie.co.uk) | £130.56 | 28 |
| Timolol Maleate Eye Drops 0.5% | £76.13 | 28 |
| Trusopt Eye Drops 2% 0.2ml Ud PF (Santen UK Limited; santen.uk) | £954.69 | 25 |
| Betnesol-N Ear/eye/nose Drops (RPH Pharmaceuticals AB; recipharm.com) | £64.82 | 25 |
| Dorzolamide 20mg/ml / Timolol 5mg/ml | £78.07 | 24 |
| Atropine 1% eye drops | £4109.00 | 23 |
| Eyeaze 0.4% eye drops PF (Ridge Pharma Ltd; ridge-pharma.com) | £116.23 | 23 |
| AaproMel 0.3% eye drops (Essential-Healthcare Ltd; essential-healthcare.co.uk) | £15.71 | 23 |
| Prednisolone Sodium Phosphate Eye Drops 0.5% Ud | £562.46 | 22 |
| Minims Cyclopentolate Hydrochloride 1% Ud PF | £223.93 | 22 |
| Carmize 0.5% Carmellose Eye Drops 10ml (NTC S.r.l; ntcpharma.com) | £167.84 | 22 |
| Doxycycline Capsule 40mg M/R | £374.61 | 21 |
| Geltears Ophthalmic Gel 0.2% (Bausch and Lomb; bausch.com) | £96.77 | 21 |
| Tiopex Gel Eye Drops 1mg/g 0.4g Ud (Thea Pharmaceuticals Ltd; thea-pharmaceuticals.co.uk) | £314.98 | 20 |
| Cetirizine 1mg/ml oral solution sugar free | £291.03 | 20 |
| Gentamicin 0.3% ear/eye drops | £346.79 | 19 |
| Eyeaze 0.2% eye drops PF (Ridge Pharma Ltd; ridge-pharma.com) | £85.33 | 19 |
| Hydrocortisone 0.5% cream | £25.02 | 19 |
| Cyclopentolate Hydrochloride Eye Drops 1% Ud | £186.13 | 18 |
| Fexofenadine 180mg tablets | £36.28 | 18 |
| Thealoz Duo Gel Sodium Hyaluronate 0.4g PF (Thea Pharmaceuticals Ltd; thea-pharmaceuticals.co.uk) | £287.03 | 17 |
| Loteprednol Etabonate Eye Drops 0.5% | £97.32 | 17 |
| Dorzolamide 20mg/ml eye drops | £88.72 | 17 |
| Lacrilube eye ointment PF (AbbVie Ltd; abbvie.co.uk) | £36.50 | 17 |
| Ibuprofen 400mg tablets | £20.45 | 17 |
| Xailin Fresh 0.5% Carmellose Eye Drops 0.4ml Ud (VISUfarma Ltd; visufarma.com) | £135.79 | 16 |
| Carmellose Sodium Eye Drops 1% 0.4ml Ud | £72.50 | 16 |
| Metronidazole Tablet 400mg | £56.25 | 16 |
| Carbomer 0.2% Eye Gel | £52.31 | 16 |
| Valaciclovir Tablet 500mg | £888.81 | 15 |
| Clarithromycin 500mg tablets | £98.91 | 15 |
| Carbomer 974p Gel Eye Drops 0.25% | £83.89 | 15 |
| Co-amoxiclav 250mg/62mg/5ml PO suspension | £79.09 | 15 |
| Liquivisc Eye Gel 0.25% (Thea Pharmaceuticals Ltd; thea-pharmaceuticals.co.uk) | £67.28 | 15 |
| Azithromycin 250mg tablets | £31.25 | 15 |
| Prednisolone Sodium Phosphate Eye Drops 1% PF | £839.66 | 14 |
| Ganfort Eye Drops 300mcg/5mg 0.4ml Ud (AbbVie Ltd; abbvie.co.uk) | £584.42 | 14 |
| Efracea Capsule 40mg M/R (Galderma Ltd; galderma.com) | £275.12 | 14 |
| Carmize 1% Carmellose Eye Drops 10ml (NTC S.r.l; ntcpharma.com) | £158.37 | 14 |
| Hyabak Sodium Hyaluronate Eye Drops (Thea Pharmaceuticals Ltd; thea-pharmaceuticals.co.uk) | £133.93 | 14 |
| Lotemax Eye Drops 0.5% (Bausch and Lomb; bausch.com) | £82.05 | 14 |
| Zeroveen Cream 100g (Thornton and Ross Ltd; thorntonross.com) | £37.48 | 14 |
| Moxisylyte Hydrochloride Eye Drops 0.5% | £127.97 | 13 |
| Chloramphenicol Ear Drops 5% | £1136.33 | 12 |
| Xailin Intense HA 0.3% eye drops PF (VISUfarma Ltd; visufarma.com) | £108.08 | 12 |
| Aactive HA PF 0.2% eye drops PF (TriOn Pharma Ltd; trionpharma.co.uk) | £52.24 | 12 |
| Lumecare Evolve 0.3% Eye Dps 10ml PF (Medicom Healthcare Ltd; medicomhealthcare.com) | £29.66 | 12 |
| Naproxen Tablet 500mg | £20.89 | 12 |
| Acetylcysteine Eye Drops 5% | £764.00 | 11 |
| Sodium Cromoglicate Eye Drops 2% 0.3ml Ud PF | £169.65 | 11 |
| Iopidine 0.5% Ophthalmic Solution 0.5% (Essential Pharma; essentialpharmagroup.com) | £161.47 | 11 |
| Aciclovir Tablet Disp 400mg | £138.63 | 11 |
| Bimatoprost 100micrograms/ml eye drops | £117.00 | 11 |
| Mydrilate Solution 0.5% (Esteve Pharmaceuticals Ltd; esteve.com) | £82.91 | 11 |
| Oxyal Sodium Hyaluronate Eye Drops (Bausch and Lomb; bausch.com) | £73.43 | 11 |
| Tear-Lac Hypromellose Eye Drops (Scope Ophthalmics Ltd; scopeeyecare.com) | £59.57 | 11 |
| Vismed Gel Sodium Eye Drops 0.45ml Ud PF (TRB Chemedica; trbchemidica.co.uk) | £346.90 | 10 |
| Bimatoprost 300micrograms/ml 0.4ml PF | £280.36 | 10 |
| Diamox Sr Capsule 250mg (ADVANZ Pharma; advanzpharma.com) | £134.99 | 10 |
| Systane Hydroxypropyl Guar Eye Drops (Alcon Eye Care UK Limited; alcon.com) | £48.34 | 10 |
| Amoxicillin 250mg/5ml oral suspension | £37.48 | 10 |
| Gppe Eye Drops Otrivine-Antistin (Thea Pharmaceuticals Ltd; thea-pharmaceuticals.co.uk) | £31.31 | 10 |
| Doxycycline Hyclate Tablet 20mg | £395.08 | 9 |
| Hydrocortisone Sodium Phosphate Eye Drops 3.35mg/ml 0.4ml PF | £173.55 | 9 |
| Dexamethasone 0.1% eye drops 0.3ml PF | £152.91 | 9 |
| Nepafenac Eye Drops 1mg/ml | £139.07 | 9 |
| HydraMed Sodium Hyaluronate Eye Drops 10ml PF (Farmigea S.p.A; farmigea.co.uk) | £69.26 | 9 |
| Alphagan Eye Drops 0.2% (AbbVie Ltd; abbvie.co.uk) | £57.24 | 9 |
| Flucloxacillin 250mg/5ml oral solution | £55.30 | 9 |
| Viscotears HA 0.1% eye drops PF (Bausch and Lomb; bausch.com) | £47.61 | 9 |
| Optho-Lique Forte 1% Carmellose 10ml (Essential-Healthcare Ltd; essential-healthcare.co.uk) | £38.65 | 9 |
| Loratadine 5mg/5ml oral solution | £29.01 | 9 |
| Sodium Chloride Eye Ointment 5% | £792.75 | 8 |
| Doxycycline Hyclate Tablet Disp 100mg | £243.51 | 8 |
| Dorzolamide 20mg/Timolol 5mg/ml 0.2ml PF | £195.51 | 8 |
| Acetazolamide Capsule 250mg | £154.38 | 8 |
| Yellox Eye Drops 900mcg/ml (Bausch and Lomb; bausch.com) | £71.34 | 8 |
| Blink Intensive Tears Sodium Hyaluronate Eye Drops 0.4ml Ud (Bausch and Lomb; bausch.com) | £39.50 | 8 |
| Carmellose Sodium Eye Drops 0.5% 0.4ml Ud | £37.32 | 8 |
| VIZhyal 0.2% eye drops PF (EM Pharma Ltd; empharma.com) | £30.67 | 8 |
| HydraMed night Parafin Eye Ointment P/F 5g (Farmigea S.p.A; farmigea.co.uk) | £22.04 | 8 |
| Doublebase Gel (Dermal Laboratories Ltd; dermal.co.uk) | £19.84 | 8 |
| Teardew 0.3% Hypromellose Eye Drops 10ml (Sai-Meds Ltd; sai-med.com) | £7.48 | 8 |
| Hydrocortisone Ointment 0.5% | £289.38 | 7 |
| Alissa 5% Sodium Chloride Eye Ointment P/F (Alissa Healthcare Research Ltd; alissahealthcare.com) | £188.48 | 7 |
| Levofloxacin 5mg/ml eye drops PF | £100.29 | 7 |
| Clinitas 0.2% eye drops 0.5ml unit dose preservative free (Altacor Ltd; altacorpharma.co.uk) | £78.17 | 7 |
| Amoxicillin 250mg capsules | £9.68 | 7 |
| NaCl 5% Sodium Chloride Eye Drops 0.45ml Ud P/F | £290.66 | 6 |
| Xalacom Eye Drops 50mcg/5ml/ml (Viatris UK Healthcare Ltd; Viatris.co.uk) | £200.11 | 6 |
| Brinzolamide/Brimonidine Eye Drops 10mg2mgml | £102.77 | 6 |
| Dorzolamide Eye Drops 20mg/ml P/F | £50.85 | 6 |
| Sofradex Ear/eye Drops (Neon Healthcare Ltd; neonhealthcare.com) | £48.83 | 6 |
| Bromfenac Eye Drops 900mcg/ml | £47.58 | 6 |
| Opticrom Eye Drops 2% (Thornton and Ross Ltd; thorntonross.com) | £44.90 | 6 |
| PF Drops 0.5% Carmellose Eye Drops 10ml | £41.94 | 6 |
| Clinitas Multi 0.2% eye drops preservative free (Altacor Ltd; altacorpharma.co.uk) | £33.55 | 6 |
| Lumecare Tear Drops Hypromellose Eye Dps 0.5ml Ud (Medicom Healthcare Ltd; medicomhealthcare.com) | £30.98 | 6 |
| Artelac Rebalance Eye Drops (Bausch and Lomb; bausch.com) | £29.82 | 6 |
| Opticrom Allergy Eye Drops 2% (Thornton and Ross Ltd; thorntonross.com) | £18.33 | 6 |
| Naproxen Tablet 250mg | £16.61 | 6 |
| Ibuprofen 200mg tablets | £9.89 | 6 |
| Prednisolone 5mg tablets | £5.02 | 6 |
| Flurbiprofen Tablet 50mg | £129.57 | 5 |
| Levofloxacin Eye Drops 5mg/ml 0.5ml Ud PF | £127.94 | 5 |
| Ivermectin Cream 10mg/g | £127.82 | 5 |
| Oxytetracycline 250mg tablets | £121.08 | 5 |
| Tacrolimus Ointment 0.1% | £95.62 | 5 |
| Nevanac Eye Drops 3mg/ml (Novartis Pharmaceuticals; novartis.com) | £83.31 | 5 |
| Sod Chloride Eye Drops 0.9% Ud | £79.58 | 5 |
| PF Drops Sodium Hyaluronate 0.4% 10ml PF | £58.65 | 5 |
| Latanoprost Eye Drops 50mcg/ml 0.2mlUd PF | £55.46 | 5 |
| Minims Sodium Chloride 0.9% Ud | £55.34 | 5 |
| Optive 0.5% Carmellose Eye Drops (AbbVie Ltd; abbvie.co.uk) | £48.80 | 5 |
| Augmentin Tab 625mg (GlaxoSmithKline; gsk.com) | £48.61 | 5 |
| Blephaclean Eye Lid Wipe (Théa Pharmaceuticals Ltd; thea-pharmaceuticals.co.uk) | £47.72 | 5 |
| Zaditen Eye Drops 250mcg/ml (Théa Pharmaceuticals Ltd; thea-pharmaceuticals.co.uk) | £43.69 | 5 |
| Diclofenac Sodium Eye Drops 0.1% | £43.48 | 5 |
| Azithromycin Oral Suspension 200mg/5ml | £35.95 | 5 |
| Co-amoxiclav 125mg/31mg/5ml PO suspension | £23.33 | 5 |
| Timoptol-La_Ophthalmic Gel-Forming Solution 0.25% (Santen UK Limited; santen.uk) | £20.43 | 5 |
| Carmize 1% Carmellose Eye Drops 0.4ml Ud (Aspire Pharma Ltd; aspirepharma.com) | £16.72 | 5 |
| Co-amoxiclav 250mg/62mg/5ml PO suspension | £10.64 | 5 |
| Simple Eye Ointment (Martindale Pharma; ethypharm.co.uk) | £361.74 | 4 |
| Iopidine 1% Ophthalmic Solution 1% Ud P/f (Essential Pharma Ltd; essentialpharmagroup.com) | £263.96 | 4 |
| Voltarol Ophthalmic Eye Drops 0.1% 0.3ml Ud (Théa Pharmaceuticals Ltd; thea-pharmaceuticals.co.uk) | £136.35 | 4 |
| Catacrom Eye Drops 2% 0.3ml Ud (Rayner Pharmaceuticals Ltd; rayner.com) | £107.51 | 4 |
| Pilocarpine Hydrochloride Eye Drops 1% P/F | £84.44 | 4 |
| Bimatoprost Eye Dps 300mcg/ml P/F | £70.80 | 4 |
| Thealoz Duo UD Sod Hyaluronate 0.4ml Ud PF (Théa Pharmaceuticals Ltd; thea-pharmaceuticals.co.uk) | £60.26 | 4 |
| MGDRx EyeBag Eye Compress (Théa Pharmaceuticals Ltd; thea-pharmaceuticals.co.uk) | £37.22 | 4 |
| Brinzolamide 10mg/ml / Timolol 5mg/ml | £33.43 | 4 |
| Voltarol Ophthalmic M/d Eye Drops 0.1% (Théa Pharmaceuticals Ltd; thea-pharmaceuticals.co.uk) | £31.01 | 4 |
| Azelastine 0.05% eye drops | £23.91 | 4 |
| Cellusan Light 0.5% Carmellose 10ml PF (Farmigea S.p.A; farmigea.co.uk) | £22.71 | 4 |
| Fexofenadine Hydrochloride Tablet 30mg | £22.16 | 4 |
| Xailin Plus Hyaluronic Acid 0.2% drops PF (Medicom Healthcare Ltd; medicomhealthcare.com) | £20.92 | 4 |
| Alomide Ophthalmic Solution 0.1% (Novartis Pharmaceuticals; novartis.com) | £19.46 | 4 |
| Flucloxacillin Sodium Oral Solution 125mg/5ml | £18.10 | 4 |
| Epaderm Cream (Mölnlycke Health Care Ltd; molnlycke.com) | £18.02 | 4 |
| Omeprazole Tablet E/C 20mg | £13.78 | 4 |
| Fusidic acid 2% cream | £9.93 | 4 |
| Azithromycin 250mg capsules | £9.09 | 4 |
| Ibuprofen 600mg tablets | £6.23 | 4 |
| Pilocarpine Hydrochloride Eye Drops 2% PF | £123.02 | 3 |
| Dorzolamide Eye Drops 2% 0.2ml Ud | £112.67 | 3 |
| Elidel Cream 1% (Bausch Health Companies Inc; bauschhealth.com) | £71.63 | 3 |
| Bimatoprost 300micrograms/ml eye drops | £51.88 | 3 |
| Desloratadine Oral Solution 2.5mg/5ml S/F | £43.69 | 3 |
| Saflutan Eye Drops 15mcg/ml (Santen UK Limited; santen.uk) | £31.88 | 3 |
| Timolol Eye Drops 2.5mg/ml P/F | £31.36 | 3 |
| Fixapost Eye Drops 50mcg/5mg/ml 0.2ml Ud (Théa Pharmaceuticals Ltd; thea-pharmaceuticals.co.uk) | £29.23 | 3 |
| Alissa Carmellose 0.5% Eye Drops 10ml (Alissa Healthcare Research Ltd; alissahealthcare.com) | £27.96 | 3 |
| Hylo-Dual Sodium Hyaluronate Eye Drops 10ml PF (URSAPHARM Arzneimittel GmbH; ursapharm.de) | £27.80 | 3 |
| Oftaquix Eye Drops 5mg/ml (Santen UK Limited; santen.uk) | £25.90 | 3 |
| Evolve Revive 0.2% eye drops PF (Medicom Healthcare Ltd; medicomhealthcare.com) | £25.18 | 3 |
| HydraMed Forte 0.4% eye drops PF (Farmigea S.p.A; farmigea.co.uk) | £21.43 | 3 |
| Timolol Maleate Gel Eye Drops 0.25% | £20.34 | 3 |
| Liquifilm 1.4% Polyvinyl Alcohol 0.4ml PF (AbbVie Ltd; abbvie.co.uk) | £19.94 | 3 |
| Fucidin H Cream (LEO Laboratories Ltd; leo-pharma.co.uk) | £16.87 | 3 |
| Hylo-Fresh Sod Hyaluronate Eye Drops PF 10ml (URSAPHARM Arzneimittel GmbH; ursapharm.de) | £13.88 | 3 |
| Phenoxymethylpenicillin 250mg tablets | £12.57 | 3 |
| VIZhyal 0.4% Sodium Hyaluronate 10ml PF (EM Pharma Ltd; empharma.com) | £11.48 | 3 |
| Amoxicillin 250mg/5ml PO suspension | £11.47 | 3 |
| Zerobase Cream (Thornton and Ross Ltd; thorntonross.com) | £11.46 | 3 |
| Aactive HA 0.1% eye drops (TriOn Pharma Ltd; trionpharma.co.uk) | £11.19 | 3 |
| Artelac Nighttime Carbomer Eye Gel (Bausch and Lomb Inc; bausch.com) | £8.31 | 3 |
| Lumecare Long Last Carbomer Eye Gel (Medicom Healthcare Ltd; medicomhealthcare.com) | £7.30 | 3 |
| Chlorphenamine Maleate Oral Solution 2mg/5ml | £6.96 | 3 |
| Aciclovir 5% cream | £5.82 | 3 |
| Exception Handler Hypromellose Item 0.3% | £5.80 | 3 |
| Co-amoxiclav 125mg/31mg/5ml PO suspension | £5.72 | 3 |
| Sno Tears 1.4% Polyvinyl Alcohol Eye Drops 10ml (Bausch and Lomb Inc; bausch.com) | £3.00 | 3 |
| Chloramphenicol Ear Drops 10% | £196.86 | 2 |
| SodiEye 5% Sod Chloride Eye Drops 0.5ml Ud P/F (TriOn Pharma Ltd; trionpharma.co.uk) | £176.11 | 2 |
| Hydrocortisone Eye Ointment 1% | £140.38 | 2 |
| Ciclosporin Eye Drops 0.1% 0.3ml Ud | £100.67 | 2 |
| Clindamycin 300mg capsules | £76.24 | 2 |
| Prednisolone Sodium Phosphate Eye Drops 0.1% | £70.65 | 2 |
| Latanoprost/Timolol Eye Drops50mcg/5mg/mlUd | £50.33 | 2 |
| Bimatoprost/Timolol Eye Drops300mcg/5mg 0.4 | £50.23 | 2 |
| PF Drops Sodium Chloride 5% Eye Drops 10ml P/F | £47.01 | 2 |
| ODM5 Sodium Chloride Eye Drops 10ml P/F (Horus Pharma; horus-pharma.com) | £44.77 | 2 |
| Protopic Ointment 0.03% (LEO Pharma; leo-pharma.co.uk) | £43.52 | 2 |
| Tacrolimus Ointment 0.03% (LEO Pharma; leo-pharma.co.uk) | £43.34 | 2 |
| Aeon 5% Sodium Chloride Eye Drops 10ml P/F (Rayner Pharmaceuticals Ltd; rayner.com) | £42.74 | 2 |
| HydraMed Sodium Hyaluronate Eye Drops 0.5ml PF (Farmigea S.p.A; farmigea.co.uk) | £41.61 | 2 |
| Ocu-Lube Carbomer 0.2% Eye Gel 0.6ml Ud P/F (Sai-Meds Ltd; sai-med.com) | £41.52 | 2 |
| Trusopt Ocumeter Plus Ophthalmic Solution 2% (Santen UK Limited; santen.uk) | £35.43 | 2 |
| Aciclovir Tablet Disp 800mg | £35.21 | 2 |
| Vibramycin-D_Tab Disp 100mg | £33.27 | 2 |
| Artelac Splash Sodium Hyaluronate 0.5ml PF (Bausch and Lomb Inc; bausch.com) | £32.65 | 2 |
| Atropine Sulphate Eye Drops 1% Ud | £29.18 | 2 |
| Minims Atropine Sulfate 1% Ud PF | £29.13 | 2 |
| EvoTears eye drops PF (URSAPHARM Arzneimittel GmbH; ursapharm.de) | £27.81 | 2 |
| Co-amoxiclav 400mg/57mg/5ml PO susp | £26.88 | 2 |
| Bimatoprost/Timolol Eye Drops 300mcg/5mg | £26.40 | 2 |
| Systane Hydroxypropyl Eye Drops 0.8ml Ud (Alcon; alconscience.com) | £25.95 | 2 |
| Erythrocin 500 Filmtab 500mg | £19.23 | 2 |
| Eykappo Eye Drops 5mg/ml (Aspire Pharma Ltd; aspirepharma.com) | £18.88 | 2 |
| Combigan Eye Drops (AbbVie Ltd; abbvie.co.uk) | £18.51 | 2 |
| Prednisolone 10mg tablets | £18.11 | 2 |
| Aeon Protect 0.3% Sodium Hyaluronate 10ml (Rayner Pharmaceuticals Ltd; rayner.com) | £17.16 | 2 |
| Telfast 30 Tab 30mg (Sanofi Winthrop Industrie; sanofi.com) | £15.28 | 2 |
| Doxycycline 100mg tablets | £15.20 | 2 |
| Vismed Sodium Hyaluronate Eye Drops 0.3ml Ud (TRB Chemedica Ltd; trbchemedica.co.uk) | £14.81 | 2 |
| Blephasol Lotion (Théa Pharmaceuticals Ltd; thea-pharmaceuticals.co.uk) | £14.39 | 2 |
| Systane Ultra Hydroxypropyl Eye Drops (Alcon; alconscience.com) | £12.64 | 2 |
| Augmentin Tab 375mg (GlaxoSmithKline; gsk.com) | £11.03 | 2 |
| Clindamycin 150mg capsules | £10.76 | 2 |
| Artelac Eye Dps 0.32% (Bausch and Lomb Inc; bausch.com) | £9.33 | 2 |
| Ciprofloxacin 500mg tablets | £9.22 | 2 |
| Xailin Hydrate Hypromellose 0.3% 10ml PF (Medicom Healthcare Ltd; medicomhealthcare.com) | £9.20 | 2 |
| E45 Cream (Karo Healthcare; karohealthcare.com) | £8.45 | 2 |
| Erythromycin Stearate 250mg tablets | £8.35 | 2 |
| Brolene Eye Drops 0.1% (Thornton and Ross Ltd; thorntonross.com) | £6.17 | 2 |
| Timoptol Eye Drops 0.25% W/v (Santen UK Limited; santen.uk) | £5.81 | 2 |
| Hydrocortisone Cream 0.1% | £5.29 | 2 |
| Naproxen 250mg gastro-resistant tablets | £5.22 | 2 |
| Brimonidine 2mg/ml eye drops | £4.78 | 2 |
| Betamethasone Sodium Phosphate Ear/eye/nsl Drops 0.1% | £4.35 | 2 |
| Chlorphenamine 4mg tablets | £4.31 | 2 |
| Predsol Ear/eye Drops 0.5% (RPH Pharmaceuticals; recipharm.com) | £4.27 | 2 |
| Eumovate Cream 0.05% (GlaxoSmithKline; gsk.com) | £3.48 | 2 |
| Lansoprazole 15mg gastro-resistant capsules | £2.49 | 2 |
| AacuLose Hypromellose 0.5% eye drops (TriOn Pharma Ltd; trionpharma.co.uk) | £1.87 | 2 |
| AaproMel 0.5% eye drops (Essential-Healthcare Ltd; essential-healthcare.co.uk) | £1.83 | 2 |
| Micropore 2.5cm X 5m Surgical Adhesive Tape | £1.75 | 2 |
| Ocufen Eye Drops 0.03% 0.4ml Ud (AbbVie Ltd; abbvie.co.uk) | £34.64 | 1 |
| Pilocarpine Nitrate Eye Drops 2% Ud (Bausch and Lomb Inc; bausch.com) | £34.61 | 1 |
| Aciclovir Oral Susp 200mg/5ml S/f | £33.35 | 1 |
| Prednisolone Sodium Phosphate Eye Drops 0.05% | £24.0049 | 1 |
| Minims Tropicamide 1% Ud P/f | £22.14 | 1 |
| Oxalux 5mg/ml eye drops 0.5ml unit dose (Kestrel Ophthalmics Ltd; kestrelophthalmics.com) | £21.33 | 1 |
| Moxivig Eye Drops 0.5% (Novartis Pharmaceuticals; novartis.com) | £18.29 | 1 |
| Prednisolone Tablet 20mg | £18.15 | 1 |
| AacEdem Sodium Chloride 5% eye drops (Essential-Healthcare Ltd; essential-healthcare.co.uk) | £16.65 | 1 |
| Cefuroxime Axetil Tab 250mg | £16.47 | 1 |
| Aeon Protect Plus 0.3% eye drops PF (Rayner Pharmaceuticals Ltd; rayner.com) | £14.54 | 1 |
| Timolol Gel Eye Drops 1mg/g 0.4g Ud P/F | £13.98 | 1 |
| Nepafenac Eye Drops 3mg/ml | £13.92 | 1 |
| Nevanac Eye Drops 1mg/ml (Novartis Pharmaceuticals; novartis.com) | £13.90 | 1 |
| DuoTrav Eye Drops (Novartis Pharmaceuticals; novartis.com) | £12.94 | 1 |
| Cetirizine Hydrochloride Capsule 10mg | £12.46 | 1 |
| Tafluprost Eye Drops 15mcg/ml 0.3ml Ud P/F | £11.39 | 1 |
| Minims Cyclopentolate Hydrochloride 0.5% Ud P/f | £11.14 | 1 |
| Hydromoor Hypromellose Eye Drops 0.4ml Ud (Rayner Pharmaceuticals Ltd; rayner.com) | £10.99 | 1 |
| Mometasone 0.1% ointment | £10.40 | 1 |
| Ocusan Sodium Hyaluronate Eye Drops 0.5ml Ud (AGEPHA Pharma s.r.o; agephapharma.com) | £10.34 | 1 |
| Travatan Eye Drops 40mcg/ml (Alcon; alconscience.com) | £10.14 | 1 |
| Zithromax Capsule 250mg (Pfizer Ltd; pfizer.com) | £9.97 | 1 |
| Erythromycin stearate 500mg tablets | £9.61 | 1 |
| Clarithromycin 250mg/5ml oral suspension | £9.31 | 1 |
| Timolol Eye Drops 5mg/ml PF | £9.01 | 1 |
| Flucloxacillin 125mg/5ml PO susp | £8.22 | 1 |
| Thealipid eye drops PF (Théa Pharmaceuticals Ltd; thea-pharmaceuticals.co.uk) | £8.12 | 1 |
| Erythromycin Ethylsuccinate Tab 500mg | £7.65 | 1 |
| Ciprofloxacin Tablet 750mg | £7.42 | 1 |
| Erythroped A Tablet 500mg | £7.29 | 1 |
| Loratadine Oral Solution 5mg/5ml S/F | £7.06 | 1 |
| Travoprost 40micrograms/ml eye drops 0.1ml PF | £6.99 | 1 |
| PF Drops 1% Carmellose Eye Drops 10ml | £6.99 | 1 |
| Eyenite 0.4% eye ointment (Warneford Healthcare Ltd; warnefordhealth.com) | £6.52 | 1 |
| Epaderm Emulsifying Ointment (Mölnlycke Health Care Ltd; molnlycke.com) | £6.21 | 1 |
| Systane Ultra Hydroxypropyl 0.7ml Ud (Alcon; alconscience.com) | £6.20 | 1 |
| Dermol Cream (Dermal Laboratories; dermal.co.uk) | £6.18 | 1 |
| Apraclonidine Eye Drops 1% Ud P/F | £6.05 | 1 |
| Optilast Eye Drops 0.05% (Mylan Products Ltd; mylan.com) | £5.94 | 1 |
| Viscotears Treha Duo eye drops PF (Bausch and Lomb Inc; bausch.com) | £5.88 | 1 |
| Clarithromycin 250mg tablets | £5.38 | 1 |
| Hypromellose 0.3% Eye Drops 10ml PF | £5.37 | 1 |
| Prednisolone Oral Solution 5mg/5ml Ud | £5.33 | 1 |
| Viscotears Hyaluronic Acid Plus 0.2% PF (Bausch and Lomb Inc; bausch.com) | £5.32 | 1 |
| Naproxen Tab E/c 500mg | £5.19 | 1 |
| Optase Moist Heat Mask Eye Compress (Scope Ophthalmics Ltd; scopeyecare.com) | £5.17 | 1 |
| Travoprost 40micrograms/ml eye drops | £5.14 | 1 |
| Cyclopentolate Hydrochloride Eye Drops 0.5% Ud | £5.13 | 1 |
| Eumovate Ointment 0.05% (GlaxoSmithKline; gsk.com) | £5.09 | 1 |
| Opticare Eye Drop Dispenser (Cameron-Graham Ltd; opticare.uk) | £4.63 | 1 |
| Cellusan 1% Carmellose Eye Drops 10ml P/F (Farmigea S.p.A; farmigea.co.uk) | £4.59 | 1 |
| Hydrocortisone Butyrate Cream 0.1% | £4.58 | 1 |
| Refresh Ophthalmic Polyvinyl Alcohol 0.4ml PF (AbbVie Ltd; abbvie.co.uk) | £4.21 | 1 |
| Zerocream (Thornton and Ross Ltd; thorntonross.com) | £4.02 | 1 |
| Ketoconazole Cream 2% | £3.97 | 1 |
| Betnovate Cream 0.1% (GlaxoSmithKline; gsk.com) | £3.78 | 1 |
| Chlorphenamine 2mg/5ml PO solution SF | £3.75 | 1 |
| Diclofenac Sodium Eye Drops 0.1% 0.3ml Ud | £3.74 | 1 |
| AaqEye Hyaluronic Acid 0.1% eye drops (Essential-Healthcare Ltd; essential-healthcare.co.uk) | £3.66 | 1 |
| Kent Pharma Sodium Hyaluronate 0.2% PF (Kent Pharma Ltd; kentathlonepharmagroup.com) | £3.27 | 1 |
| Ibuprofen Capsule 200mg | £3.10 | 1 |
| Hydromol Ointment (Alliance Pharmaceuticals Ltd; alliancepharmaceuticals.com) | £3.03 | 1 |
| Timoptol-La Ophthalmic Gel-Forming Solution 0.5% (Santen UK Limited; santen.uk) | £2.92 | 1 |
| Piriton Syrup 2mg/5ml (Haleon UK Trading Ltd; haleon.com) | £2.60 | 1 |
| Amoxicillin 125mg/5ml oral suspension | £2.59 | 1 |
| Beclometasone 50micrograms nasal spray | £2.56 | 1 |
| Fucidin Ointment 2% (LEO Laboratories Ltd; leopharma.co.uk) | £2.51 | 1 |
| Zerodouble Gel (Thornton and Ross Ltd; thorntonross.com) | £2.19 | 1 |
| Cefalexin Capsule 250mg | £2.12 | 1 |
| Fucidin Cream 2% (LEO Laboratories Ltd; leopharma.co.uk) | £1.79 | 1 |
| Xylometazoline Hydrochloride Nasal Drops Paed 0.05% | £1.78 | 1 |
| Tears Naturale Eye Drops (Alcon; alconscience.com) | £1.76 | 1 |
| Eyeaze Carmellose 1% eye drops PF (Ridge Pharma Ltd; ridge-pharma.com) | £1.70 | 1 |
| Cetraben Cream 50g (Thornton and Ross Ltd; thorntonross.com) | £1.39 | 1 |
| AaCarb Carbomer 0.2% eye gel (TriOn Pharma Ltd; trionpharma.co.uk) | £1.28 | 1 |
| Aacomer 0.2% eye gel (Essential-Healthcare Ltd; essential-healthcare.co.uk) | £1.26 | 1 |
| Puroptics Carbomer 0.2% eye gel (Biovantic Pharma Ltd; biovanticpharma.co.uk) | £1.22 | 1 |
| Diprobase Cream 50g (Bayer PLC; bayer.co.uk) | £1.20 | 1 |
| Opticrom Aqueous (Thornton and Ross Ltd; thorntonross.com) | £0.86 | 1 |
| Hypromellose Eye Drops 0.5% | £0.76 | 1 |
| **Total** | **£339,426.48** | **49162** |

***Supplement 1****. List of items prescribed by independent prescribing optometrists in Wales from 2020-2024.*
